# Supplementary material for: The Negative Impact of Sarcopenia on Hepatocellular Carcinoma Treatment Outcomes
Source: Cancers (Basel). 2024 Jun 24;16(13):2315. doi: 10.3390/cancers16132315 (PMC11240545; doi:10.3390/cancers16132315)
Supplement: Supplementary file 1 [file cancers-16-02315-s001.zip › cancers-2974540-supplementary.pdf]

Supplementary Materials.

Table S1. General characteristics of studies evaluating the impact of sarcopenia on liver transplant and surgical resection outcomes.

First Author and year	Number of patients enrolled	Gender (% of male)	Etiologies of HCC	Evaluation of sarcopenia	Percentage of sarcopenic patients	HCC treatment outcomes related to sarcopenia*
**LIVER TRANSPLANT**						
**Retrospective studies**						
Beumer BR (2022) [30]	528	86%	HBV/HCV/alcoholic/NASH/HDV/cryptogenic/others	SMI at CT scan	33%	Reduced OS (HR 1.44, CI 95% 1.07-1.94)
Tan Y (2022) [31]	70	100%	HBV/others	PMI at CT scan	54%	Reduced OS (HR 3.22, CI 95% 1.15-8.98)<br>Reduced RFS not statistically significant (HR 2.6, CI 95% 0.95-7.1)
Acosta LF (2019) [34]	119	80%	HCV/alcoholic	SMI at CT scan	51%	Prolonged length of hospital stays after transplant
Itoh S (2016) [32]	153	56%	HCV/others	SVR at CT scan	25%	Reduced OS (HR 2.58, CI 95% 1.17-5.52)<br>Reduced RFS (HR 5.3, CI 95% 2.03-13.8)
Valero V (2015) [33]	96	61%	HBV/HCV/others	Total psoas volume and area at CT scan	49%	Higher risk of postoperative complications (40 vs 18%, HR 3.1, CI 95% 1.07-8.72)<br>No association with OS
**SURGICAL RESECTION**						
**Prospective studies**						
Hu J (2022) [132]	153	87%	HBV/others	SMI at CT scan, hand grip, chair stand test, and gait speed	29%	Higher mortality rate in sarcopenic vs non-sarcopenic at one year (9% vs 1%, p=0.04)
Yang J (2022) [38]	155	87%	Not specified	SMI at CT scan, hand grip, chair stand test, and gait speed	15% low muscle mass *and* strength<br>42% low muscle mass *or* strength	Higher risk of major surgical complications (HR 10.7, CI 95% 2.55-45.24)
**Retrospective studies**						
Beumer BR (2023) [40]	553 (174 Dutch, 379 Japanese)	76%	HCV/HBV/others	SMI at CT scan	Not specified	Reduced OS in Japanese patients (HR 2, CI 95% 1.3-3.08) but not in Dutch patients (HR 0.76, CI 95% 0.42-1.36)
Hayashi H (2023) [46]	303	73%	HBV/HCV/others	SMI at CT scan	35%	Reduced OS (HR 1.66, CI 95% 1.00-2.76)
Kong Q (2023) [133]	112	81%	NASH	SMI at CT scan	34%	Reduced OS (HR 4.09, CI 95% 1.23-13.6)<br>Reduced RFS (HR 2.5, CI

						95% 1.42-4.49)
Wu DH (2023) [134]	1172	Not specify	HBV/others	SMI at CT scan	36%	Reduced OS (HR 1.8, CI 95% 1.57-2.16)<br>Reduced RFS (HR 1.58, CI 95% 1.36-1.84)<br>Higher risk of postoperative complications (HR 1.78, CI 95% 1.35-2.33)
Harimoto N (2022) [135]	157	83%	HCV/others	SMI and PMI at CT scan	Not specified	No association with OS or RFS
Meister FA (2022) [35]	100	72%	Viral/alcoholic /NASH/others	SMI and skeletal muscle attenuation at CT scan	54% sarcopenia 60% myosteatosis	No association with OS or disease-free survival
Kim H (2022) [136]	159	84%	Viral/others	SMI at CT scan	46.5%	Reduced OS (HR 2.1, CI 95% 1.09-4.14)<br>No association with RFS
Marasco G (2022) [37]	159	80.5%	Viral/alcoholic /NASH/others	SMI at CT scan	52%	Higher risk of major surgical complications in sarcopenic vs non-sarcopenic (12% vs 0%, p=0.03)
Jang HY (2021) [137]	160	75%	HBV/HCV/others	PMI, psoas muscle attenuation and VATI at CT scan	17.5%	Reduced OS (HR 2.2, CI 95% 1.02-4.94) (low PMI)<br>Reduced OS (HR 1.77, CI 95% 1.04-3.02) (high VATI)<br>No association with RFS
Liao C (2021) [42]	452	85%	HBV/others	SMI at CT scan	18% sarcopenic obesity	Reduced OS (HR 3.0, CI 95% 2.24-4.08) (sarcopenic obesity)<br>Reduced RFS (HR 1.98, CI 95% 1.5-2.6) (sarcopenic obesity)
Seror M (2021) [36]	110	84%	NASH	SMI at CT scan	24%	Higher risk of postoperative complications (HR 6.5, CI 95% 2.08-20.4)<br>Lower RFS (17 vs 61 months, p= 0.03) in sarcopenia with high LSN vs non sarcopenia and high LSN<br>No association with OS
Hamaguchi Y (2019) [45]	606	80%	HBV/HCV/others	SMI and IMAC at CT scan	14% Low SMI 43%high IMAC	Reduced OS (HR 1.3, CI 95% 1.01-1.75) (SMI)<br>Reduced RFS (HR 1.5, CI 95% 1.07-1.91) (SMI)<br>Reduced OS (HR 1.8, CI 95% 1.37-2.3) (IMAC)<br>Reduced RFS (HR 1.2, CI 95% 1.001-1.49) (IMAC)
Kobayashi A (2019) [41]	465	79%	HBV/HCV/others	SMI and VATI at CT scan	7% Sarcopenic normal weight	Reduced OS (HR 2.5, CI 95% 1.34-4.5, 84.7 vs 39.1 months, p=0.002) (sarcopenic obesity)

					7% sarcopenic obesity	Reduced RFS (HR 2.03, CI 95% 1.23-3.22, 21.4 vs 8.4 months, p=0.003) (sarcopenic obesity)
Hiraoka A (2018) [138]	171	76%	HBV/HCV/alcoholic/others	Psoas muscle area at CT scan	20.5% with muscle loss	Reduced OS (HR 1.9, CI 95% 1.05-3.42) (muscle loss)
Harimoto N (2016) [139]	296	75%	HBV/HCV/others	SMI at CT scan	38%	Reduced OS (HR 2.5, CI 95% 1.21-5.59)<br>Reduced RFS (HR 1.8, CI 95% 1.04-3.19)
Kamachi S (2016) [57]	92	71%	HCV	SMI at CT scan	66%	Higher risk of HCC recurrence (HR 1.89, CI 95% 1.03-3.46)
Kobayashi A (2016) [44]	241	80%	Not specified	PMI and IMAC at CT scan	Not specified	The increase risk of HCC recurrence (HR 3.7, CI 95% 1.18-13.5)
Takagi K (2016) [39]	254	82%	HBV/HCV/others	SMI at CT scan	46.5%	Reduced OS rate (HR 2.28, CI 95% 1.34-4.01, 58% vs 82%, p=0.0002)<br>Poorer ASA status (HR 3.17, CI 95% 1.61-5.94)
Yabusaki N (2016) [140]	195	80%	HBV/HCV/others	SMI and VFA at CT scan	46%	Increase HCC recurrence (HR 1.6, CI 95% 1.1-2.5) (BMI≥22 kg/m2)
Levolger S (2015) [55]	90	70%	HBV/HCV/others	SMI at CT scan	58%	Reduced OS (HR 3.8 CI 95% 1.78-7.93, 33 vs 105 months p=0.002)<br>Higher treatment-mortality (17% vs 3%, p=0.029)<br>Higher rate of major surgical complications (33 vs 13%, p=0.03)
Valero V (2015) [33]	96	61%	HBV/HCV/others	Total psoas volume and area at CT scan	Not specified	Higher risk of postoperative complications (HR 3.1 CI 95% 1.19-7.63, 40% vs 18% p=0.01).<br>No association with long-term survival
Voron T (2015) [141]	109	84%	HBV/HCV/alcoholic/NASH/multifactorial/unknown	SMI at CT scan	54%	Reduced OS (HR 3.2 CI 95% 1.28-7.96, 52.3 vs 70.3 months p=0.015)
Itoh S (2014) [43]	190	77%	Not specified	SMI and VFA at CT scan	Not specified (sarcopenia) 44% low VFA	Reduced OS (HR 1.96, CI 95% 1.06-3.74) (SMI)<br>No association with RFS
Harimoto N (2013) [142]	186	78%	Not specified	SMI at CT scan	40%	Reduced OS (HR 0.9, CI 95% 0.84-0.96, 71% vs 84% at 5 years p=0.001)<br>Reduced RFS (HR 0.97, CI 95% 0.95-1.00, 13% vs 33% at 5 years p=0.013)

*All data were obtained through multivariate analysis.

ASA: American Society of Anesthesiology. CT: computed tomography. HBV: hepatitis B virus. HCC: hepatocellular carcinoma. HCV: hepatitis C virus. HDV: hepatitis D virus. HR: hazard ratio. IMAC: intramuscular adipose tissue content. LSN: liver surface nodularity. MVL: muscle volume loss. NASH: nonalcoholic steatohepatitis. OS: overall survival. PMI: psoas muscle index. RFS: recurrence-free survival. SMI: skeletal muscle index. SVR: skeletal muscle mass-to-visceral fat area ratio. VATI: visceral adipose tissue index. VFA: visceral fat area.

Table S2. General characteristics of studies evaluating the impact of sarcopenia on locoregional treatment outcomes.

First Author and year	Number of patients enrolled	Gender (% of male)	Etiologies of HCC	Evaluation of sarcopenia	Percentage of sarcopenic patients	HCC treatment outcomes related to sarcopenia*
**ABLATION TREATMENT**						
**Prospective studies**						
Nakai M (2022) [54]	160	59%	HCV/others	SMI at CT scan	22%	Reduced OS (HR 3.34, CI 95% 1.19-9.37) (only in HCV-negative patients)
Salman A (2021) [53]	97	74%	HCV	SMI at CT scan	43%	Reduced OS (HR 7.6, CI 95% 3.1-18.7)
**Retrospective studies**						
Jaruvongvanich V (2023) [52]	56	71%	HBV/HCV/others	SMI at CT scan	66%	No significant association with HCC recurrence (HR 2.06, CI 95% 0.99-4.27)
Yeh WS (2020) [56]	136	57%	HBV/HCV/others	PMI at CT scan	16%	Reduced OS of 20% at 5 years (HR 2.47, CI 95% 1.3-4.7)
Kamachi S (2016) [57]	92	66%	HCV	SMI at CT scan	66%	Higher HCC recurrence (HR 1.89, CI 95% 1.03-3.46)
Levolger S (2015) [55]	90	70%	HBV/HCV/others	SMI at CT scan	58%	Reduced OS (33 vs 105 months, p=0.002)
**TACE TREATMENT**						
**Prospective studies**						
Hashida R (2020) [73]	152	64%	HBV/HCV/NASH/alcoholic/AIH/others	SMI at CT scan	Not specified	No association with OS
**Retrospective studies**						
Bannangkoon K (2023) [78]	611	73%	HBV/HCV/alcoholic/others	SMI and SMD for myosteatosis at CT scan	32% sarcopenic, 39% with myosteatosis	Reduced OS (HR 1.26, CI 95% 1.04-1.52) (sarcopenia)<br>Reduced OS (HR 1.66, CI 95% 1.37-2.01) (myosteatosis)
Li Y (2023) [76]	235	74%	Not specified	SMI and VFI at CT scan	60% sarcopenic, 46% high VFI	Reduced OS (HR 5.7, CI 95% 3.6-9.1) (sarcopenia)<br>Reduced OS (HR 3.4, CI 95% 2.2-5.3) (high VFI)
Loosen SH (2023) [62]	89	69%	HBV/HCV/NASH/alcoholic/ot	SMI at CT scan	Not specified	Reduced OS (HR 0.90, CI 95% 0.83-0.98)

			hers			
Wang S (2023) [63]	364	82%	HBV/HCV	SMI at CT scan	58%	Reduced OS (HR 1.6, CI 95% 1.21-2.14)
Chien TP (2022) [68]	260	74%	HBV/HCV	PMI at CT scan	50%	Reduced OS (HR 1.36, CI 95% 1.003-1.85, 18 vs 25 months p=0.011)
Masetti C (2022) [79]	151	77%	HBV/HCV/NASH/ alcoholic/rare	SMI at CT scan IMAC at CT scan for myosteatosis	85% sarcopenia 76% myosteatosis	No differences in OS
Roth G (2022) [69]	225	89%	NASH/HCV/HBV	SMI and PMI at CT scan	58%	Reduced OS (19.4 vs 35.5 months, p=0.015) Reduced PFS (8.3 vs 13.2 months, p=0.0035)
Zhang JX (2022) [65]	228	77%	HBV/others	SMI and PMI at CT scan	46% low SMI 39% low PMI	Reduced OS (15.8 vs 33.5 months, p<0.001) (SMI) Reduced OS (16.9 vs 38.5 months, p<0.001) (PMI)
Lim J (2021) [75]	266	70%	HBV/HCV/others	SMI and VSR at CT scan	30% sarcopenic obesity	Reduced OS (HR 1.5, CI 95% 0.66-1.11)
Sugama Y (2021) [67]	87	70%	HBV/HCV/others	PMI at CT scan	Not specified	Reduced OS (HR 0.67, CI 95% 0.49-0.92)
Zheng X (2021) [71]	75	84%	Not specified	Cross sectional area of paraspinal muscles at CT scan	61% skeletal muscle loss	Reduced OS (HR 5.9, CI 95% 2.46-12.3) (skeletal muscle loss)
Lanza E (2020) [77]	142	77%	HBV/HCV/NASH/alcoholic	SMI at CT scan	85%	Reduced OS (HR 2.22, CI 95% 1.01-4.86)
Fujita M (2019) [72]	179	73%	HBV/HCV/NASH/alcoholic/others	PMI at CT scan	45%	Reduced OS (HR 1.88, CI 95% 1.3-2.7) (change in PMI)
Loosen SH (2019) [66]	46	79%	Not specified	PMI at CT scan	Not specified	Reduced OS (16.4 vs 43 months, p=0.017)
Kobayashi T (2018) [70]	102	69%	HBV/HCV/NASH/alcoholic/rare	SMI at CT scan	30%	Reduced OS (HR 1.67, CI 95% 1.03-2.72) (skeletal muscle loss)
Dodson RM (2013) [64]	216	64%	HBV/HCV/others	PMI at CT scan	32% sarcopenic normal weight 15% sarcopenic obesity	Reduced OS (17.0 vs 33.3 months, p=0.02 in men; 15.8 vs 22.1 months, p=0.81 in women)
**RADIOTHERAPIES**						
**Prospective studies**						
Saalfeld S (2023) [87]	150	88%	HBV/HCV/alcohol/NASH/AIH /rare	SMI, SAT, VAT at CT scan	Not specified	Reduced OS at 1-year (sarcopenia)
**Retrospective studies**						
Yang JF (2022) [83]	137	78%	HBV/HCV/others	SMI at CT scan	49%	Reduced OS (HR 1.96, CI 95% 1.15-3.33, 6.3 vs 31.4 months p<0.001) (skeletal muscle loss)

Guichet PL (2021) [86]	82	79%	HBV/HCV/alcoholic	Paraspinal muscle mass at MRI	30%	Reduced OS (HR 1.94, CI 95% 1.01-3.74, 8.9 vs 31.9 months p<0.001)
Vallati GE (2021) [88]	86	76%	Not specified	PMI at CT scan	49%	High time-to-progression (HR 3.25, CI 95% 1.35-7.81)
Faron A (2020) [85]	58	78%	HBV/HCV/alcoholic/others	Paraspinal muscle mass at MRI	50%	Reduced OS (HR 2.675, CI 95% 1.25-5.7, 197 vs 294 days p=0.02) Tendence to reduce PFS (109 vs 185 days, p=0.068)
Lee J (2019) [84]	156	82%	HBV/HCV/others	SMI at CT scan	63%	Reduced OS baseline sarcopenia (HR 2.38, CI 95% 1.53-3.7, 7.1 vs 15.3 months p<0.001) (baseline sarcopenia) Reduced OS (HR 2.5, CI 95% 1.28-5.02, 14.1 vs 17.5 months p=0.02) (post therapy sarcopenia)
Shiba S (2018) [89]	68	60%	Not specified	SMI at CT scan	32%	No association with OS and PFS

*All data were obtained through multivariate analysis.

AIH: autoimmune hepatitis. CT: computed tomography. HBV: hepatitis B virus. HCC: hepatocellular carcinoma. HCV: hepatitis C virus. HR: hazard ratio. IMAC: intramuscular adipose tissue content. MVL: muscle volume loss. MRI: magnetic resonance imaging. NASH: nonalcoholic steatohepatitis. OS: overall survival. PMI: psoas muscle index. PFS: progression-free survival. RFS: recurrence-free survival. SMD: skeletal muscle density. SMI: skeletal muscle index. TACE: transarterial chemoembolization. VAT: visceral adipose tissue. VFI: visceral fat index. VSR: visceral to subcutaneous fat area ratio.

Table S3. General characteristics of studies evaluating the impact of sarcopenia on systemic treatment outcomes.

First Author and year	Number of patients enrolled	Gender (% of male)	Etiologies of HCC	Evaluation of sarcopenia	Percentage of sarcopenic patients	HCC treatment outcomes related to sarcopenia*
**TKI**						
**Prospective studies**						
Saalfeld S (2023) [87]	147	86%	HBV/HCV/alcohol/NASH/AIH/rare	SMI, SAT, and VAT at CT scan	Not specified	No association with OS
**Retrospective studies**						
Imai K (2023) [102]	77	86%	HBV/HCV/others	SMI, SATI, and VATI at CT scan	25% had rapid SMI reduction after treatment	Reduced OS (HR 0.94, CI 95% 0.91-0.98) (sarcopenia) Reduced OS (15.7 vs 23.1 months, p<0.001) (skeletal muscle loss)
Dong D (2022) [96]	40	92.5%	HBV/HCV/others	SMI at CT scan	57.5%	Reduced OS (HR 0.26, CI 95% 0.08-0.79, 8.4 vs 14.7 months p=0.02).

						No association with PFS
Fujita M (2022) [103]	130	82%	HBV/HCV/alcoholic/NASH/others	PMI at CT scan	48.5%	Reduced OS (HR 1.93, CI 95% 1.06-3.5, 15.2 vs 25.6 months p=0.005) (skeletal muscle loss)
Toshida K (2022) [109]	63	68%	HBV/HCV/others	SMI at CT scan	63%	Reduced OS (HR 2.86, CI 95% 1.11-7.33).<br>No association with PFS
Hiraoka A (2021) [97]	151	77%	HBV/HCV/alcoholic/others	PMI at CT scan	27%	Reduced OS (HR 1.65, CI 95% 1.02-2.69, 27.9% vs 46.1% at 1.5 years p<0.001)<br>Reduced PFS (4.5% vs 11.6% at 1.5 years p=0.025)<br>Higher risk of severe adverse events AEs (44% vs 18%, p=0.003)
Sugama Y (2021) [67]	87	70%	HBV/HCV/others	PMI at CT scan	Not specified	Reduced OS (HR 0.67, CI 95% 0.49-0.92)
Wu CH (2021) [98]	137	88%	HBV/HCV/others	Skeletal muscle mass at CT scan	15% (in males)	Reduced OS (HR 1.73, CI 95% 1.06-2.83) (males)
Cheng TY (2020) [105]	385	78%	HBV/HCV/alcoholic/others	PMI at CT scan	65%	Reduced PPS (HR 1.34 CI 95% 1.06-1.68, 3.8 vs 5.8 months p=0.003)
Endo K (2020) [106]	63	84%	HBV/HCV/alcoholic/others	SMI at CT scan, grip strength	35% reduced SMI<br>33% reduced grip strength	Reduced OS (HR 3.55, CI 95% 1.42-8.92) (grip strength)<br>No association with OS and PFS (baseline sarcopenia)
Uojima H (2020) [101]	100	75%	HBV/HCV/alcoholic/NASH/others	SMI at CT scan	59%	Reduced OS (HR 2.25, CI 95% 1.09-4.62, 264 vs 353 days p=0.021)<br>Reduced TTF (HR 1.72, CI 95% 1.04-2.83, 139 vs 230 days p=0.01)
Imai K (2019) [104]	61	88.5%	HBV/HCV/others	SMI, VFMI and SFMI at CT scan	Not specified	Reduced OS (HR 4, CI 95% 1.8-8.9) (SMI)<br>Reduced OS (HR 4.1, CI 95% 1.97-8.58) (SFMI)
Antonelli G (2018) [95]	96	78%	HBV/HCV/alcohol/NASH/others	SMI at CT scan	49%	Reduced OS (HR 1.6, CI 95% 1.05-2.53, 39 vs 61 weeks p=0.01)<br>Shorter time on treatment (HR 1.7, CI 95% 1.12-2.64, 12 vs 26 weeks p=0.004)
Takada H (2018) [92]	214	78%	Not specify	SMI at CT scan	57%	Reduced OS (HR 1.6, CI 95% 1.0-2.4, 417 vs 562 days p=0.047)
Hiraoka A (2017) [94]	93	87%	HBV/HCV/others	PMI at CT scan	21.5%	Reduced OS (HR 2.2, CI 95% 1.07-4.3, 32% vs 48% at 1.5 years p=0.04)<br>No association with PFS
Nishikawa H (2017) [93]	232	78%	HBV/HCV/others/unknown	SMI at CT scan	65%	Reduced OS (HR 0.36, CI 95% 0.25-0.52, 174 vs 454 days p<0.0001)<br>Reduced tumour response rate (ORR 4% vs 13.6%, p=0.015)

						No association with PFS
Yamashima M (2017) [99]	40	92.5%	HBV/HCV/others	PMI at CT scan	Not specified	Reduced OS (HR 2.3) (skeletal mass loss)<br>No association with OS or PFS (baseline sarcopenia)
Mir O (2012) [100]	40	75%	HBV/HCV/alcoholic/others	SMI at CT scan	27.5%	Higher drug toxicity (82% vs 31%, p=0.005)
**ICI**						
**Retrospective studies**						
Chen BB (2023) [118]	138	87%	HBV/HCV/others	SMI at CT scan for sarcopenia, mean muscle attenuation at CT scan for myosteatosis	46% sarcopenia<br>15% myosteatosis<br>7% sarcopenic obesity	Reduced OS (HR 2.1, CI 95% 1.29.3.88, 13.8 vs 27.7 months p=0.002) (sarcopenia)<br>No association with PFS (sarcopenia)<br>Reduced OS (HR 2.2, CI 95% 1.23-3.88, 4.3 vs 18.7 months p<0.001) (myosteatosis)<br>Reduced PFS (HR 2, CI 95% 1.15-3.5, 1.6 vs 5.3 months p<0.001) (myosteatosis)<br>No association with OS and PFS (sarcopenic obesity)
Hiraoka A (2023) [108]	525	80%	HBV/HCV/alcoholic/others	SMI at CT scan	37.5% muscle loss	Correlation between muscle loss and GNRI (AUC 0.715).<br>Reduced OS (HR 1.445, CI 95% 1.16-1.8) (patients with decline in GNRI)
Oura K (2023) [110]	64	77%	HBV/HCV/alcoholic/NASH/PBC	SMI at BIA	44%	Reduced OS (HR 2.87, CI 95% 1.29-6.4)<br>Reduced PFS (4.7 vs 10.6 months, p<0.05)<br>Higher rate of AEs (89% vs 67%, p<0.05)
Scheiner B (2023) [113]	176	81%	Viral/alcoholic/others/unknown	PMI at CT scan	33%	Reduced OS (HR 1.63, CI 95% 1.07-2.48, 7.2 vs 22.6 months p<0.001)<br>Reduced PFS (HR 1.54, CI 95% 1.06-2.23, 3.4 vs 7.9 months p=0.001)<br>Reduced tumour response rate (22% vs 39%, p=0.03)
Xiong B (2023) [117]	74	85%	HBV/others	SMI, TATI, SATI, and VATI at CT scan	53%	Reduced OS S (HR 2.2, CI 95% 1.1-4.3) (sarcopenia)<br>Reduced PFS (HR 2.38, CI 95% 1.33-4.23) (sarcopenia)<br>Reduced OS (HR 0.25, CI 95% 0.11-0.58) (SATI)
Guo Y (2022) [114]	97	81%	HBV/others	SMI at CT scan	47%	Reduced PFS (HR 0.51, CI 95% 0.3-0.86, 4.8 vs 6.5 months p=0.038)<br>No association with OS

Matsumoto H (2022) [107]	32	59%	HBV/HCV/alcoholic/NASH	SMI at CT scan	44%	Reduced PFS (HR 5.1, CI 95% 1.0-21.4, 5.8 vs 8.5 months p=0.011) (skeletal muscle loss)<br>No association with PFS (baseline sarcopenia)
Toshida K (2022) [109]	35	80%	HBV/HCV/others	SMI at CT scan	57%	No association with OS and PFS
Zhao M (2022) [112]	160	81%	HBV/others	PMI at CT scan	75%	Reduced OS (HR 2.46, CI 95% 1.31-4.64, 19.7 vs 29.1 months p=0.001)<br>Reduced PFS (HR 1.76, CI 95% 1.09-2.83, 4.1 vs 8.5 months p=0.003)
Kim N (2021) [115]	102	85%	HBV/HCV/alcoholic/others	SMI at CT scan	22.5%	Reduced OS (2.9 vs 7.5 months, p=0.036)<br>No association with PFS and disease progression rate

*All data were obtained through multivariate analysis.

AEs: adverse events. CT: computed tomography. GNRI: geriatric nutritional risk index. HBV: hepatitis B virus. HCC: hepatocellular carcinoma. HCV: hepatitis C virus. HR: hazard ratio. ICI: immune checkpoint inhibitor. NASH: nonalcoholic steatohepatitis. OS: overall survival. PMI: psoas muscle index. PFS: progression-free survival. PPS: post-progression survival. RFS: recurrence-free survival. SATI: subcutaneous adipose tissue index. SFMI: subcutaneous fat mass index. SMI: skeletal muscle index. TATI: total adipose tissue index. TKI: tyrosine kinase inhibitors. TTF: time to treatment failure. VATI: visceral adipose tissue index. VFMI: visceral fat mass index.
